# A game-based learning approach to sleep hygiene education: a pilot investigation

**DOI:** 10.3389/fdgth.2024.1334840

**Published:** 2024-04-12

**Authors:** Christine Seaver, Clint Bowers, Deborah Beidel, Lisa Holt, Sridhar Ramakrishnan

**Affiliations:** ^1^ UCF RESTORES, University of Central Florida, Orlando, FL, United States; ^2^ BlueHalo, Rockville, MD, United States

**Keywords:** sleep hygiene, game-based learning, serious games, games for health, sleep hygiene education (SHE)

## Abstract

**Introduction:**

Sleep hygiene education (SHE) consists of environmental and behavioral practices primarily intended to reduce sleep problems. Currently considered ineffective as a stand-alone treatment, the manner in which the education is typically delivered may be ineffective for the acquisition of new knowledge. The purpose of this study was to determine if a more engaging teaching medium may improve the efficacy of sleep hygiene education. This study examined the use of game-based learning to teach SHE to individuals with sleep problems.

**Methods:**

35 participants played the SHE games for 30 days. Differences in pre- and post-state anxiety and sleep quality measures were examined.

**Results:**

Participants had significant improvements in sleep quality and state anxiety after using the app for 30 days, although scores for the majority of patients remained elevated.

**Discussion:**

This pilot investigation provides initial evidence for the efficacy of a game-based approach to SHE.

## Introduction

Quality sleep is imperative for health and overall quality of life. In fact, dimensions of sleep quality have been linked to numerous poor health outcomes such as coronary heart disease and hyptertension in numerous cross-sectional (1, 3) and cohort (4) studies. These studies generally demonstrate a moderate effect of sleep quality on health outcomes. Cross-sectional (5) and experimental (6) studies have also demonstrated relationships between sleep problems and increased levels of anxiety. More specifically, one study (7) found that individuals reported significanly lower sleep quality on days when they experienced higher levels of stress. Similarly, another study (8) found that stressful life events had a direct effect on sleep quality. These findings are not surprising given that poor sleep quality leads to a physiological neuroendocrine stress response in which sympathetic tone and cortisol levels are increased (9). Finally, research has shown that reduced sleep quality can bave impacts beyond physical and mental health. Indeed, a cross-sectional study indicated that poor sleep leads to occupational impairments, such as reduced productivity and efficiency (10).

Two primary indicators of good sleep quality are (1) a sleep latency of ≤15 min and (2) sleeping through the night (or only having few awakenings of ≤5 min) (11). However, a recent National Health Interview (12) found that over a 30 day period, 14.5% of adults in the United States had difficulty falling asleep and 17.8% of adults staying asleep. These problems were more prevalent depending on factors such as race, socioeconomic status, urbanization level, age, and biological sex (12). In particular, the report found that individuals with lower levels of education and income had more trouble falling asleep. Additionally, non-hispanic white adults had more trouble staying asleep compared to non-hispanic black, Hispanic, and non-hispanic Asian adults. Furthemore, those in more rural areas had more trouble falling and staying asleep. Given this host of negative outcomes, a significant percentage of the population could benefit from evidence-based interventions to improve sleep.

Primary care providers (PCP) are often the first point of contact for individuals presenting with difficulties falling or staying asleep (13). A retrospective chart review of the patients who discussed sleep problems with their PCP found that 51.5% of the sample were offered a sleep medication. Intensive Cognitive Behavior Therapy was recommended to 5% of the patients, whereas sleep hygiene education (SHE) was provided to 31.5% of the patients (14). Sleep hygiene consists of recommendations that optimize sleep (15, 16), and a review (13) included caffeine intake, tobacco use, alcohol intake, napping, wake/sleep time, and exercise as the most covered topics. These recommendations are typically delivered verbally or printed on a handout (17). One survey (18) revealed that although 97% of general practitioners utilized SHE as a management tool for their patients, they often perceived SHE alone to be ineffective. Prior to concluding that SHE alone is ineffective, examining whether an alternative delivery method that would optimize acquisition and implementation of appropriate sleep behaviors merits consideration.

### Delivering sleep hygiene education via gaming

SHE requires the acquisition and practice of certain behaviors, but typically is delivered in a single meeting with a practitioner. Such formats may not encourage engagement or provide opportunities to review and practice the behaviors. An alternative format is game-based learning, where learners interact with challenging interactive activities with a clear set of goals, constraints, and rules (19). Game-based learning formats offer more engaging learning mediums, and opportunities to practice newly acquired skills within a realistic context. Additionally, gaming environments afford the learner more control and feedback over traditional digital training, which, in turn, can increase motivation (19, 21). Finally, the “play” factor of games may increase motivation to learn (22–24), enhance educational achievement in comparison to a traditional learning environment (25, 26). As such, play can engage individuals in the process of learning, and thereby lead to better outcomes. Given its numerous advantages, it is not surprising that game-based learning has been on the rise over the last several decades. Games are used for learning in several industries, including mental health (27), healthcare (28), and training (29). In particular, serious game interventions have been shown to improve sleep quality in children. A study by Wilson, Miller, Bonuck, Lumeng, and Chervin (2014) (30) the “Sweet Dreamzzz Early Childhood Sleep Education Program™” “Dreamzzz Early Childhood Sleep Education Program™”. One component of this program utilized game-based learning to promote sleep hygiene. Although the study did not parse out the efficacy of each individual component, the intervention led to improvements in sleep duration. Another study by Almondes and Leonardo (2019) (31) evaluated the effects of a serious game called “Perfect Bedroom: learn to sleep well” in children. The study found small but positive changes in the sleep routines of those in the experimental group. In perhaps the only study that addressed clinical insomnia, adults who engaged in cognitive training games (e.g., Sudoku) over 15-days had improved cognitive performance, mood, and changes in sleep quality (32). These data suggest that games are efficacious in promoting knowledge and skill acquisition, providing support for the testing of this format to teach SHE.

In summary, sleep problems are common in the general population and most people initially seek help from their PCP. Pharmacological agents are most commonly offered but the most common non-pharmacological intervention is SHE (14), which is considered only minimally effective, with effect sizes no larger than those found for psychological placebo (33). Although superior psychological treatments for sleep problems (e.g., CBT-I) exist, it is probable that many individuals never get referred, or are unable to pay, for this type of specialized treatment (14). Thus, many patients may be limited to receiving SHE as typically delivered in one office encounter with no follow-up. This manner of delivery is inconsistent with how acquisition of new knowledge and skills are acquired and maintained. One question is therefore whether delivery of SHE in a different format, such as game-based learning, may improve learning and thereby, its efficacy at relieving sleep difficulties. As such, this pilot study examines the use of a game-based sleep hygiene application in improving sleep quality.

### Hypotheses

1. Sleep problems as assessed by scores on the Pittsburgh Sleep Quality Index (PSQI) will decrease (indicating better quality sleep) from pre-assessment to post-assessment.
2. Self-reported state anxiety as assessed by the State-Trait Anxiety-State (STAI-S) scores will decrease (indicating lower levels of state anxiety from pre-assessment to post-assessment).

## Methods

### Inclusion and exclusion criteria

Participants were at least 18 years old and reported trouble sleeping as assessed by scores ≥5 on the Pittsburgh Sleep Quality Index (PSQI) (34) at pre-assessment. Scores of ≥5 on the PSQI have been previously validated as indicative of poor sleep and is a commonly used cutoff score (34–36). Participants were included regardless of whether they were taking sleep medications.

### Recruitment

This study was approved by the University of Central Florida's Institutional Review Board (IRB). Flyers were posted on community boards throughout Orange County, Florida. A link to the digital version of this flyer was included in a weekly email sent to the University of Central Florida community. Interested individuals contacted the researcher to receive an informed consent document. The signed consent was returned prior to beginning the study.

### Demographics

Of the 35 participants, 5 declined to answer the demographic questionnaire. Demographics of the remaining 30 participants can be found in Table 1.

**Table 1:** Participant demographics.

Age	35.31 (SD = 16.25)
Female	63.4%
Male	36.6%
White/Caucasian	60%
Black	25%
Asian	5%
Pacific Islander	0%
Other	10%

## Materials and procedure

### Intervention description

Restful Journey is a mobile application designed to teach SHE and apply that knowledge in several play scenarios. Developed by the University of Central Florida (UCF) and Intelligent Automation, a Blue Halo Company, Restful Journey contains a series of mini-games that teach and reinforce good sleep hygiene by rewarding users for achieving sleep goals. The sleep hygiene concepts covered throughout the game were based on the American Academy of Sleep Medicine's (AASM) guidance (37). See Table 2 for a list of sleep hygiene behaviors taught in Restful Journey.

**Table 2:** Sleep hygiene concepts taught in restful journey.

Napping for 30 min or less (or not at all).
Getting at least 30 min of physical activity.
Keeping the bedroom temperature between 63 and 66 degrees.
Waking up at the same time as the previous day.
Going to bed at the same time as the previous day.
Engaging in an appropriate activity before bedtime (e.g., journaling, yoga, reading, sex).
Having last caffeine intake 4–6 h before bed (or not at all).
Having last alcohol intake at least 4–6 h before bed (or not at all).
Having last meal or snack at least 3 h before bed.
Falling asleep within 20 min.
If not falling asleep within 20 min, doing an appropriate activity.
If getting up due to not falling asleep- waiting until tired to go back to bed.
Keeping the lights dim or off prior to going to bed.
Only engaging in sleep and/or sex in bed.
Getting 7–9 h of sleep.

Restful Journey encompasses a sailing exploration theme. Players begin by learning sleep hygiene in preparation for their “journey”. Over time as they progress throughout the game (i.e., achieve sleep hygiene goals) they are able to explore different islands and obtaining jewels from each.

“Healthy Habits” is the first mini-game. This is a quiz-based game comprised of three levels. Players learn SHE through playing the game and receiving feedback regarding their responses. Players must accurately answer 90% of questions to proceed to the next level. Each level increases in difficulty by decreasing the time allotted to answer the questions. In keeping with the theme of Restful Journey, the overarching goal of this game is to teach the “sailor” (i.e., the user) on how to stay well-rested in preparation of their journey.

“Sleep Scrutiny” is the second mini-game in which players progress to “Sleep Captain”. Players must inspect their crewmate's quarters and identify objects that are conducive or detrimental to sleep. Again, there are three levels and rooms must be inspected with 100% accuracy to move forward. The time given to identify objects and answer questions decreases with each level. In keeping with the story of Restful Journey, players must help their crewmates stay well-rested to ensure that the crew is ready to set sail.

Participants were asked to dedicate 10–15 min per day to playing these first two games (i.e., “Healthy Habits” and “Sleep Scrutiny”), with the goal of completing them with a 90% correct score by the end of the first week of the study.

The final game, “Vigilant Voyage” translates game-based learning into real-life. Each day, players open the game and indicate which sleep hygiene behaviors they used the previous day. Coins are awarded for each behavior. Once the check-in is completed, the game generates a random sleep goal. If the player achieved that goal, they received bonus coins. Once enough coins are earned, the player can explore various islands and obtain jewels. Players must cash-in their coins to explore an island, requiring them to continue earning coins each day by meeting sleep hygiene goals to continue exploring other islands. Players “win” the game by collecting all of the jewels.

The game is structured to encourage all participants to meet the same sleep hygiene goals. Additionally, the game-based learning aspect provides motivation to work on any unmet goals.

**Figure 1:**
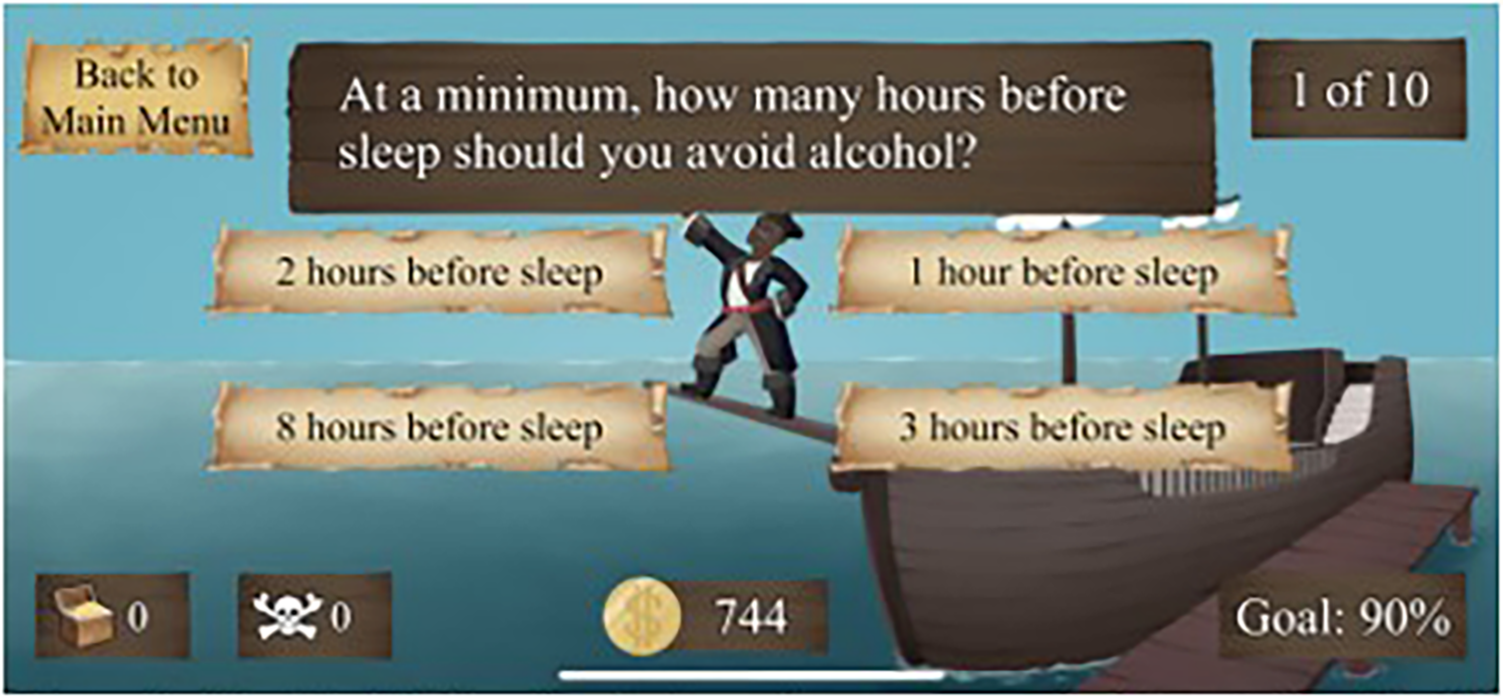
Healthy Habits. A Quiz-based game to teach players sleep hygiene principles.

**Figure 2:**
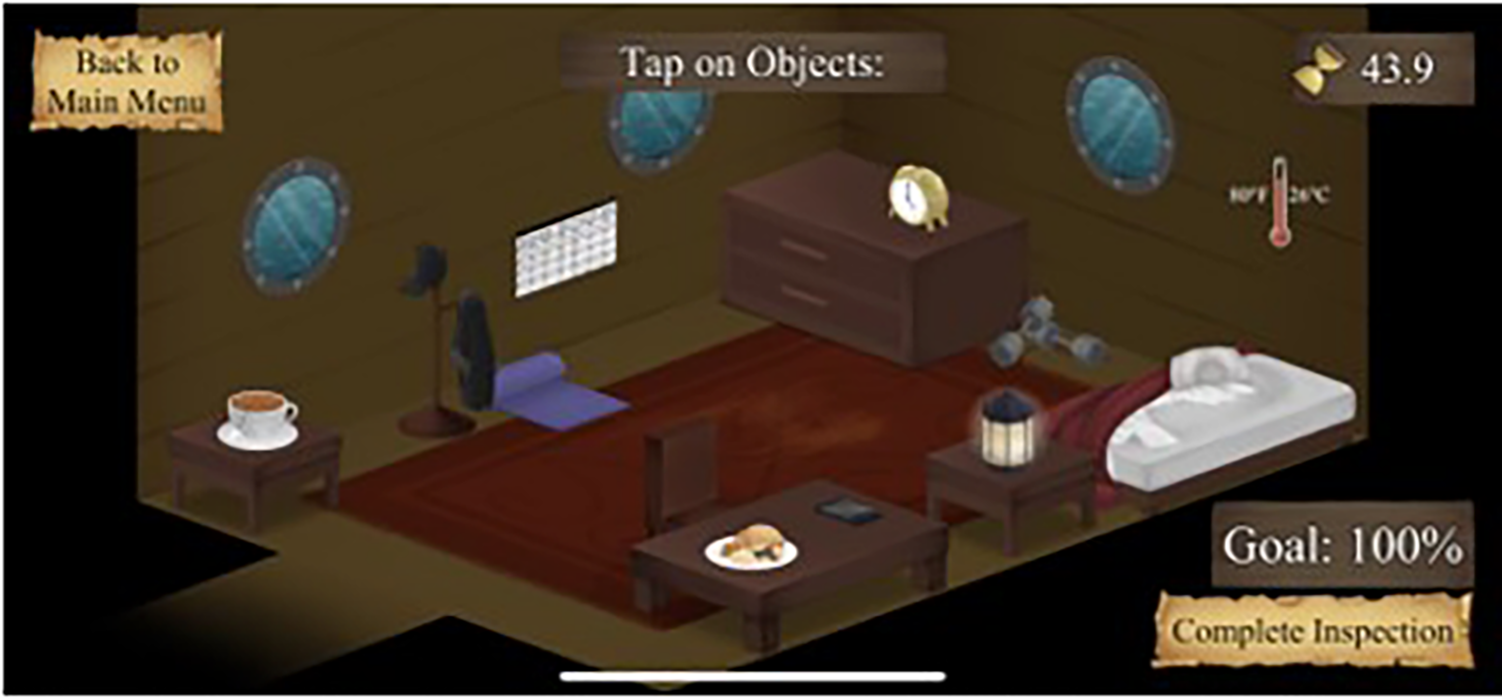
Sleep Scrutiny. Players prepare virtual bedroom for sleep.

**Figure 3:**
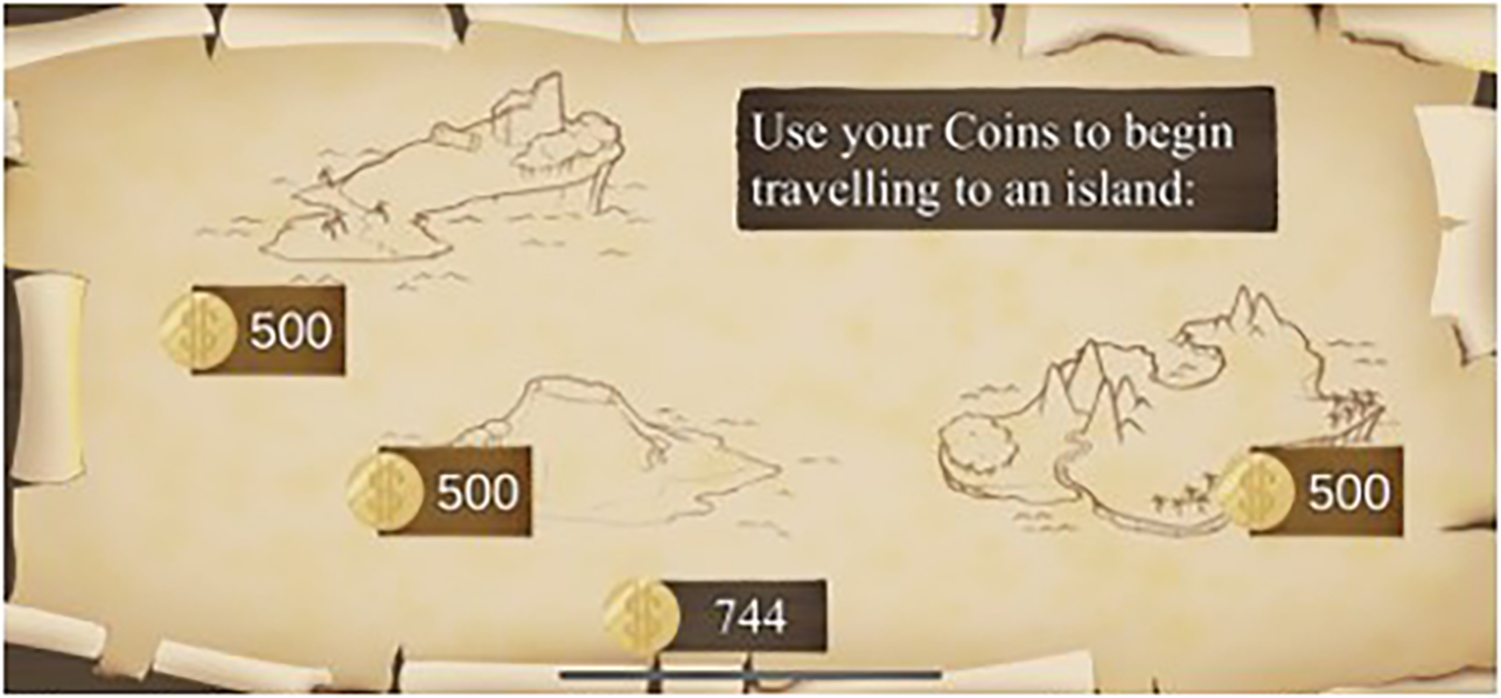
Vigilant Voyage. Players progress along the map as they earn points for meeting sleep goals.

**Figure 4:**
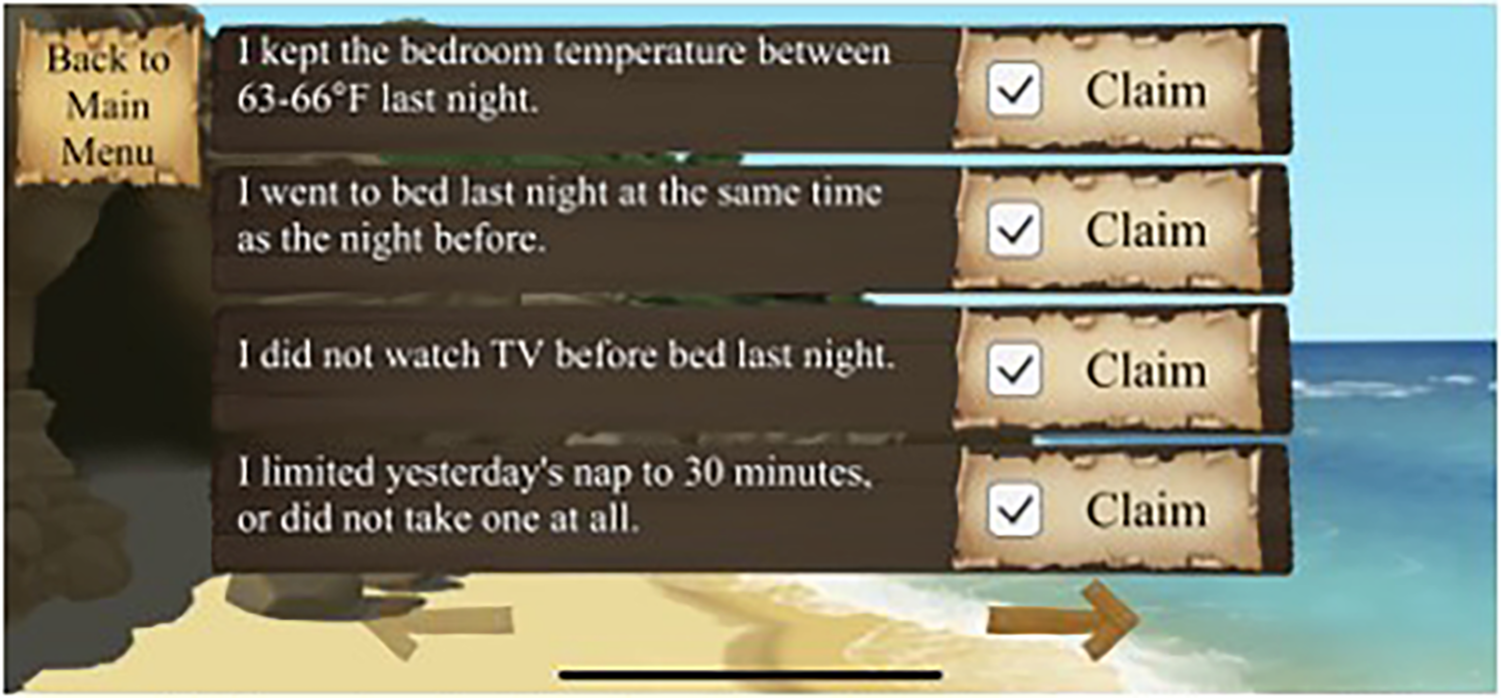
Players log in daily to claim which sleep goals they met the previous day.

See Figures 1–4 for visual depictions of Restful Journey.

### Assessment measures

Assessment measures were completed at pre and posttreatment. The assessment included:

• The Pittsburgh Sleep Quality Index (PSQI) (34) is a self-report measure of sleep quality. The PSQI distinguishes “good” and “poor” sleepers. This assessment measures seven dimensions of sleep quality: (1) subjective sleep quality, (2) sleep latency, (3) sleep duration, (4) sleep efficiency, (5) sleep disturbance, (6) use of sleeping medication, and (7) daytime dysfunction. These components can be looked at individually or added together to achieve a “global score of sleep quality.” Global PSQI score above a “5” indicates “poor sleep quality.” It should be noted that a score above a “5” does not necessarily indicate a particular clinical sleep disorder. This assessment was chosen to measure sleep quality due to its robust psychometric properties (38–40). The PSQI is the most frequently used generic measure of sleep quality among clinicians and researchers (40). As such, many studies have used the PSQI to measure improvements in sleep quality following an intervention (41–44).
• The State-Trait Anxiety Inventory (STAI-S) (45) is a widely used measure of state anxiety (i.e., current feelings of anxiety). Participants indicate the extent of their agreement with several items on a 4-point scale. The scale is internally consistent (*α* = .90). Higher means of the sum of scores indicate higher degrees of anxiety. THE STAI has been used to evaluate the treatment of medical and anxiety disorders in thousands of studies (46) and is considered to be an adequate measure of anxiety in research and clinical settings.

After completing the screening and pre-assessment, participants received instructions to download Restful Journey on their smartphone. It was downloadable on Android or iPhone devices.

Participants were emailed a daily check-in questionnaire each day, which tracked the sleep hygiene goals achieved the previous night, which game(s) they played, and the time spent playing the game. The purpose of the daily check-in was twofold; (1) assess which sleep hygiene behaviors changed over the course of the study and (2) to ensure that participants were interacting with the game daily throughout the study duration. All participants completed at least 90% of the daily check-ins. Upon completion of the post-assessment (which was identical to the pre-assessment) participants received a digital $25 Amazon gift card.

### Power analysis

An *a priori* power analysis was conducted using G*Power version 3.1 (47) for sample size estimation. The primary outcome measure was the PSQI (36). According to the power analysis, which was conducted for a paired samples *t*-test using an effect size of .5, and a significance criterion of *α* = .05, the sample size needed to achieve a power of .85 is 31. An effect size of .5 was chosen as it is generally considered to be a moderate effect size (48) and is therefore sufficient to demonstrate practical significance of the results. Thus, the obtained sample size of *n* = 35 is more than adequate to test the study hypotheses.

## Results

### App usage

Based on the Daily Check-In data, participants self-reported spending an average of 9.16 min per day (SD = 7.32) playing Restful Journey. The average number of days played was 28.4. All participants reported that they completed all mini-games and the game in its entirety.

### Sleep quality and state anxiety

All data analyses were conducted using paired sample *t*-tests. Due to a substantial number of tests (*n *= 9), we applied a false discovery rate correction to calculate adjusted *p* value thresholds for each test (see Table 3). The pre and post-scores for each of the dependent variables are displayed in Table 3. The results indicated that Restful Journey resulted in statistically significant improvements on several aspects of sleep quality assessed by the PSQI, including: global sleep quality, subjective sleep quality, sleep latency, slepe duration, daytime dysfunction, sleep efficiency, and sleep disturbance. The average score on the PSQI at post-assessment was 7.03. After playing Restful Journey for 30 days, 22.8% of participants had PSQI global scores of <5 at the post-assessment, meaning that these individuals no longer met the criteria for being “poor sleepers”. The results also indicated that Restful Journey resulted in statistically significant improvement of state anxiety as measured by the STAI.

**Table 3:** Assessment scores and Benjamin and Hochberg false discovery rate correction.

Test no. (ranked in order by size of *p* value)	Variable	Mean pre-test score	Mean post-test score	Effect size (*d*)	Estimated *p* value^a^	Corrected threshold^b^	Outcome
1	PSQI- Global score	10.54 (*SD *= 3.25)	7.03 (*SD *= 3.34)	1.06	<.001	.005	Significant
2	PSQI- Subjective sleep quality	1.69 (*SD *= .63)	1.03 (*SD *= .66)	.91	<.001	.011	Significant
3	PSQI- Sleep latency	2.26 (*SD *= .82)	1.66 (SD = .87)	.71	<.001	.016	Significant
4	PSQI- Sleep duration	1.34 (SD = .76)	.77 (SD = .73)	.94	<.001	.022	Significant
5	PSQI- Daytime dysfunction	1.51 (SD = .78)	.91 (SD = .70)	.74	<.001	.027	Significant
6	PSQI- Sleep efficiency	1.49 (SD = 1.17)	.94 (SD = 1.03)	.55	.002	.033	Significant
7	STAI- State anxiety	38.46 (SD = 11.66)	35.14 (SD = 10.83)	.39	.026	.038	Significant
8	PSQI- Sleep disturbance	1.46 (SD = .70)	1.20 (SD = .58)	.39	.027	.044	Significant
9	PSQI- Medication usage	.71 (SD = 1.10)	.68 (SD = 1.18)	.36	.831	.05	Not significant

^a^ Estimated *p* value refers to *p* value produced by each of the individual tests performed.

^b^ Corrected threshold refers to the multiple comparison corrected α level, the metric to which *p* values are compared.

## Discussion

The overarching aim of this paper was to examine the utility of game-based learning to deliver SHE and examine its impact on numerous subcomponents of sleep quality, as well as state anxiety. This pilot investigation provides modest initial evidence for the use of *Restful Journey* to improve multiple facets of sleep quality as defined by the PSQI. In particular, these improvements were reported on the subcomponents of subjective sleep quality, sleep latency, and sleep duration. Moreover, participants had a significant improvement in sleep efficiency, another subcomponent of the PSQI. There was also a significant improvement in sleep disturbance. Finally, there was a significant decrease in daytime dysfunction.

Although participants experienced a statistically significant improvement in overall sleep quality as well as six of its seven components, the average score on the PSQI at post-assessment was above “5”, indicating that participants were still having some problems with sleep. Additionally. participants did not significantly decrease their use of sleep medication, as measured by a subcomponent of the PSQI. The daily check-in data indicated that the participants were using the app almost every day, so adherence to playing the game was not an issue. In other words, the participants played the game daily, thus simply providing more opportunities for knowledge acquisition does not explain SHE's limited efficacy.

When placing these results in context within the larger literature, there are relatively few studies that have investigated the efficacy of sleep hygiene interventions in nonclinical samples. Overall, this small body of work provides some preliminary support for the use of sleep hygiene education in nonclinical populations, but the findings are inconsistent. Sleep hygiene education did not significantly improve sleep quality in adolescents (49, 50) or a sample of IT employees (51). On the other hand, sleep hygiene education was successful in improving sleep quality in athletes (52, 53), female workers (54), and women with HIV or AIDS (55). Restful Journey produced effects more in line with the latter studies demonstrating modest improvements in sleep quality.

Although outside the scope of the present study, differences in SHE efficacy among these different populations may suggest an effect of individual differences (56). In the present study, nearly one quarter of participants had PSQI global scores of <5 at the post-assessment, indicating a positive response to playing the game in these individuals. Although the personal characteristics that might predict who would positively respond to game-based learning are not known at this time, this percentage indicates that a subset of the general population might respond to a game-based application, a concept sometimes known as “person-centered care”. In fact, a unique strength of serious games is the ability to implement machine learning (57), thus allowing the game to tailor SHE to each individual's unique needs. Future work may examine whether individual differences are indeed predictive of SHE outcomes, and whether tailoring goals and learning outcomes through the implementation of machine learning algorithms into game-based SHE interventions may yield better results. Physicians generally agree that sleep quality decreases with increased age (58). In fact, an epidemiological survey of over 9,000 senior individuals found that more than 80% had at least one complaint related to their sleep quality (59). Thus, future work should also seek to identify whether age may moderate the relationship between SHE and sleep quality. Finally, although its efficacy in terms of improving sleep quality appears to be comparable to traditional SHE approaches, perhaps it boils down to personal preference; the game format of Restful Journey may appeal to certain individuals who may not be motivated to engage with traditional SHE formats. The “play” factor behind Restful Journey may make it more appealing and therefore potentially increase the uptake of SHE, even if the outcomes are ultimately similar to those of traditional SHE.

Finally, state anxiety scores also significantly decreased from pre to posttreatment after playing *Restful Journey.* Previous links between sleep difficulties and anxiety have established that these conditions are often comorbid and have a bidirectional predictive relationship (60). Both problems have mental health implications. Therefore, it was hypothesized that state anxiety would improve after playing Restful Journey, which was supported, as participants had a significant decrease in state anxiety from pre- to post- assessment. This study is consistent with previous research that has demonstrated that improved sleep quality can lessen state anxiety.

## Limitations

As a pilot investigation, this study has several limitations. The most obvious limitation is that it was not a randomized controlled trial and therefore did not have a control group. Comparing Restful Journey to the traditional form of SHE and a waitlist control group would help provide further evidence for the notion of a game-based SHE format. Another limitation of the present study is that there was no assessment for underlying sleep disorders among participants. In the general population, sleep disorders are largely undiagnosed and the disorder is often complex. There are a number of reasons for this underdiagnosis; (1) limited training in the recognition of sleep disorders, (2) an uncertainty of how to treat sleep disorders, and (3) the patient and/or provider fails to mention sleep issues (61). It is therefore possible that some study participants could have more serious sleep disorders, such as sleep apnea, which may limit the extent to which SHE alone can improve their sleep quality. Additionally, it is unknown whether participants may have sought other sleep interventions during the study. Therefore, we cannot be certain if the effects could be attributed to other interventions or treatments. Finally, this study was limited to thirty days of app usage. It is not known (1) if longer use of the app may have resulted in continued improvement in sleep or (2) whether the significant improvements were stable several months later. Future investigations should address these issues.

### Contributions and future directions

Despite these limitations, the present study found initial evidence for a game-based SHE format. The nature of the game-based interventions circumvents a number of barriers that individuals face when seeking relief for sleep problems; including ease of access, ease of use, minimal financial cost, and extensive opportunities to learn and practice these skills/behaviors in an engaging environment. The present study also demonstrated that state anxiety significantly decreased. Given that trouble sleeping can exacerbate anxiety (62), this decrease in anxiety is likely due to the improvement in sleep quality. As nearly one quarter of participants no longer met the criteria for being “poor sleepers” after using Restful Journey for 30 days, we propose that Restful Journey may be a tool for some individuals with non-clinical sleep problems to improve their sleep quality. Those that do not experience a significant improvement in sleep should seek more intensive treatment. Likewise, the addition of this application to more intensive clinical treatments may yield optimal results.

Future work may examine if the game may also reduce other mental health issues by improving overall sleep quality. Furthermore, future studies may seek to compare Restful Journey to the traditional method of SHE, which is typically a simple informational sheet. Finally, future work may examine the use of Restful Journey in combination with more intensive treatments (e.g., CBT-I) to improve treatment efficacy. Doing so would also provide insight as to whether game-based SHE is just as effective as the present gold-standard approach to alleviating sleep problems.

## Data Availability

The datasets presented in this article are not readily available because due to the funding source, there are restrictions that apply to the sharing of data. Reasonable requests will be considered on an individual basis. Please contact the corresponding author, CS, Ph.D. at christine.seaver@ucf.edu. Requests to access the datasets should be directed to christine.seaver@ucf.edu.
